# Does Eligibility Classification Matter? Tracking Cardiac Autonomic Function during a Collegiate Soccer Season

**DOI:** 10.3390/sports9060074

**Published:** 2021-05-25

**Authors:** Rohan Edmonds, Rowan Kraft, Melissa Cantu, Elizabeth Meister, P. J. Huynh, Scott Bankers, Jacob Siedlik

**Affiliations:** 1 Department of Exercise Science & Pre-Health Professions, Creighton University, 2500 California Plaza, Omaha, NE 68111, USA; RowanKraft@creighton.edu (R.K.); MelissaCantu@creighton.edu (M.C.); ElizabethMeister@creighton.edu (E.M.); P.J.Huynh@creighton.edu (P.J.H.); 2 Athletic Performance, Creighton University, 2500 California Plaza, Omaha, NE 68111, USA; ScottBankers@creighton.edu

**Keywords:** heart rate variability, athlete monitoring, team sport, college sport

## Abstract

The current study examined differences in heart rate (HR) variability (HRV) across student-athlete eligibility classifications within a men’s soccer team. The study also aimed to identify any differences in HRV while competing at home or away. Data collection covered an entire collegiate season, commencing in the preseason and concluding upon elimination from the NCAA Soccer tournament. Comparisons of HR and HRV, paired with self-reported subjective measures, were documented between student-athlete eligibility classifications, home versus away games, and based on soccer position (forward, midfielder, defender, goalkeeper). HR and HRV were similar based on student-athlete eligibility. Heart rate exhibited a small, but statistically significant decrease (β = −1.7 bpm (95% CI: −2.9, 0.57), *p* = 0.003) for the away games relative to home. HRV showed a statistically significant increase in the away game setting (β = 2.1 (95% CI: 0.78, 3.38), *p* = 0.002). No difference in HRV was observed across eligibility classification. This lack of difference may be attributed to a different perception of stress amongst male athletes. Athletes also exhibited a reduced HRV at home, likely as an indication of their readiness to compete paired with an increased self-confidence, given there was no difference in any subjective measures of mood or stress or between games played away or at home.

## 1. Introduction

Participating in sport and education is highly stressful [1,2,3,4]. In particular, collegiate student-athletes are susceptible to experiencing multiple stressors from both their sporting and academic commitments [5]. Student-athletes are required to balance the demands of class, practice, competition, and their own personal life on a continuous basis [1]. While sport involvement has been shown to alleviate stress [6,7], research also suggests that athletic participation itself can become an additional stressor amongst collegiate student-athletes [6,8]. Further, student-athletes regularly experience unique stressors that accompany their athletic status such as competition-related stressors like injury concerns, or the possibility of limited playing time or having to sit out a season, and/or organizational stressors, including potential conflicts with teammates or coaching staff [2]. Specifically, freshman athletes who are familiarizing themselves with the increased academic and competitive workloads associated with their first year in college may carry a greater physiological and psychological workload [8]. In contrast, student-athletes in their final year of eligibility observe reduced stress levels, with the experience gained over their collegiate career helping them better manage their time, academic and sporting workload [9]. Despite it being well known that student-athletes face a myriad of stressors, less is known regarding how collegiate athletes manage the added stress of a congested match schedule and the travel that accompanies collegiate sport.

Heart rate (HR) variability (HRV) may provide a means to help better understand the impact of both the psychological and physiological demands placed on student-athletes. Defined as the variability between successive heart beats, HRV offers a useful non-invasive estimate of cardiac autonomic function [10]. A practical option due to its non-invasiveness and time-efficiency [11], HRV has been shown to be a beneficial means when examining psychological health and stress across a variety of situations [12,13,14,15]. In particular, research has identified an inverse relationship between HRV and perceived emotional stress [15], and also work-related stress [13]. These studies suggest that an increased perception of emotional and work stressors is often coupled with a reduction in cardiac autonomic function within an adult population. In a sporting environment, HRV provides coaches and athletes practical information regarding potential long-term training adaptations, while also identifying an athlete’s readiness to train [16,17,18]. Periods of intense training or high-intensity exercise have been shown to reduce HRV for up to 48 h [19,20,21], with recovery to resting HRV typically faster in well-trained individuals [20].

It is worthwhile to note that fluctuations in HRV deemed unfavorable, and suggestive of fatigue, may be instigated by reasons unrelated to sport-specific training and competition demands [22]. As previously mentioned, stress [12] and other factors such as mood state and sleep quality may also alter HRV patterns [23,24]. Given student-athletes face a multitude of various stressors, integrating subjective measures of athlete wellness may be of benefit for coaches and support staff to better understand and manage student-athlete health and wellbeing.

Considered the most popular sport in the world [25], soccer is characterized as a high-intensity, intermittent non-continuous sport [25,26]. At an elite level, the distance covered during gameplay varies greatly, ranging from 8–14 km [25,27,28]. While most distance is covered at a low intensity [28], research suggests distances covered at a high intensity are more valid measures of physical performance as they are mostly closely associated with training status [29]. The average cardiovascular workload during a typical 90-min match ranges between 80 and 90% HR_max_, with little to no difference between professional and non-professional soccer [26]. Research has also shown that the recovery kinetics of soccer are dependent on the number of sprints and hard changes of direction performed during a match, and that time motion analysis may be of benefit in helping identify post-match fatigue [30]. Likewise, research suggests an active recovery regime, as opposed to soccer-specific training sessions, promotes enhanced recovery following competitive soccer games [31].

The overarching purpose of the current study was to examine the differences in HRV across student-athletes of different eligibility classifications. An additional aim of the study was to document potential differences in HRV across soccer positions, and when competing at home or away.

## 2. Materials and Methods

Conducted over the entirety of a collegiate soccer season, this longitudinal study documented the cardiac vagal activity in a squad of division I National Collegiate Athletic Association (NCAA) soccer players. Comparisons were made between athletes across different eligibility classifications (Freshman, Sophomore, Junior, Senior). Additional comparisons were analyzed between games competed at home or away, and between days (pre-gameday, gameday, post-gameday, and pre/post gameday), with pre/post gamedays considered a day where the team had a game the day prior to a recording/a game the day after a recording. Lastly, comparisons were also analyzed between the different soccer positions (Forward, Midfield, Defense, Goal Keeper).

### 2.1. Participants

Student-athlete characteristics are listed in Table 1. Seventeen male soccer players between the ages of 19 and 22 provided voluntary written informed consent and were enrolled in the study. Criteria for inclusion were established to ensure that all recruited athletes were current members of the university’s soccer program, had competed competitively at the high school level, and had a minimum of three years competitive soccer experience. Athletes were excluded from the study if their HR recording compliance dropped below 80% or if they incurred any injury that resulted in consecutive missed training sessions or consecutive missed games. Ethics approval was obtained prior to the commencement of data collection from the institutional review board at the university (Study #2000381). All athletes understood the possible risks involved with participation, were advised they were free to withdraw at any time during the data collection period, and completed written informed consent prior to participating in the study.

### 2.2. Data Collection

Data collection commenced in late August and finished in early November. The data collection period encompassed a total of 20 games (13 home and 7 away) and included preseason training up until the team’s final game of the season in the conference championship. Prior to the start of data collection, skinfold thickness was measured to determine body fat percentage, with measurements recorded at three anatomical landmarks (chest, abdomen, and mid-thigh) to the nearest 5 mm and on the right side of the body. Body fat percentage was then estimated using the Jackson and Pollock equation for men [32].

As per previously established guidelines [16], a one-minute (55-s recording preceded by a 5-s stabilization period) HR and HRV (the natural log of the root mean square of successive differences between normal heartbeats; lnRMSSD) measurement was recorded each morning after waking and bladder emptying. While in a seated position, a 60 s (5 s stabilization and 55 s recording) measurement was recorded via an infrared pulse finger sensor (ithlete; HRV Fit Ltd. Southampton, United Kingdom) connected to a smartphone application (ithlete HRV). When prompted by the application, athletes followed a paced breathing instruction (7.5 breaths per minute) to ensure uniformity of measurement. The lnRMSSD value was multiplied by 20 for easier interpretation, allowing for a ~100-point scale for analysis. Any artifacts or ectopic beats were removed by an in-built algorithm within the application. After completing the HR measurement, athlete self-reported measures (ASRM) of sleep quality, fatigue, muscle soreness, stress, and mood state were recorded by means of a visual analog scale (9 = best rating and 1 = worst rating).

### 2.3. Statistical Analysis

Due to the nested data structure and unbalanced experimental design (e.g., unequal number of players at each eligibility level and/or position group), dependent variables were analyzed via multilevel regression models using the lme4 package in R version 4.0.3. Independent variables were modeled as fixed effects, while intercepts were modeled as random effects for each athlete. Estimates and 95% confidence intervals (CI) are reported with estimates interpreted as statistically significant if the 95% CI does not include zero. All visual representations of the data are presented as mean ± standard deviation. Data were found to be normally distributed prior to analysis and an alpha-level of *p* < 0.05 was used for all statistical analyses.

## 3. Results

### 3.1. Anthropometrics

Athletes across different eligibility classifications and positions were of similar (*p* > 0.05) height, weight, and body composition (Table 1).

### 3.2. Student-Athlete Eligibility and Position

**Figure 1:**
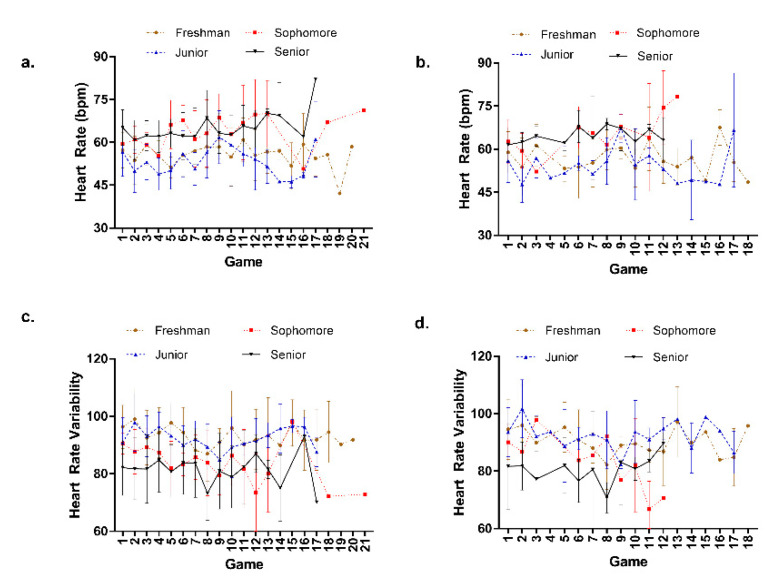
Mean (±standard deviation) heart rate ((**a**)—the morning of each game; (**b**)—the morning after each game) and heart rate variability ((**c**)—the morning of each game; (**d**)—the morning after each game) for each eligibility classification (total n = 17).

There were no significant differences in HR or HRV based on student-athlete eligibility over the course of the season (Figure 1, summary data shown in Table 2). Similarly, there were no significant differences observed across the eligibility levels when examining the ASRM subjective indices. Likewise, there were no significant differences in HR or HRV based on position. There were also no differences in subjective indices of the ASRM across positions.

### 3.3. Home vs. Away

**Figure 2:**
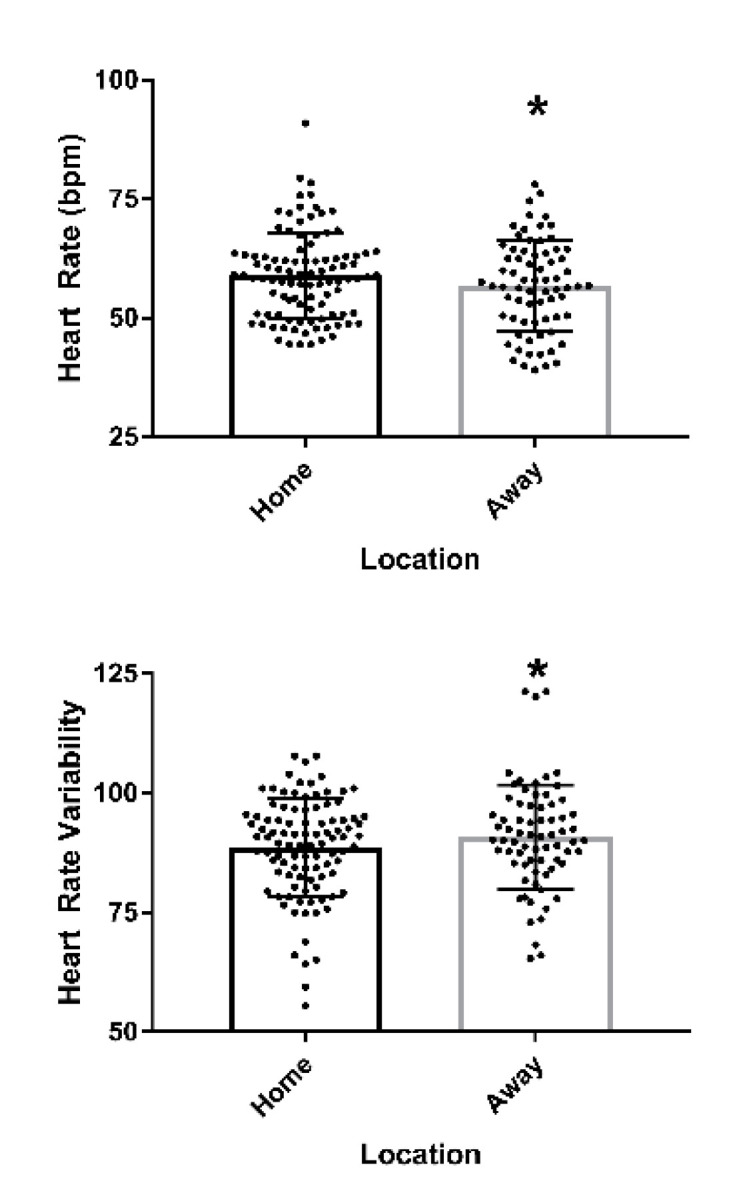
Mean (± standard deviation) heart rate and heart rate variability for the entire squad (n = 17) for games played at home and away. Dots represent individual measures of heart rate and heart rate variability. * indicates *p* < 0.05 compared to home.

Heart rate exhibited a small, but statistically significant decrease (β = −1.7 bpm (95% CI: −2.9, 0.57), *p* = 0.003) for the away games relative to home. HRV showed a statistically significant increase in the away game setting (β = 2.1 (95% CI: 0.78, 3.38), *p* = 0.002). Muscle soreness also exhibited a small, but statistically significant decrease when playing away (β = −0.26 (95% CI: −0.5, −0.02), *p* = 0.03) (Figure 2).

### 3.4. Pre vs. Post Game

**Figure 3:**
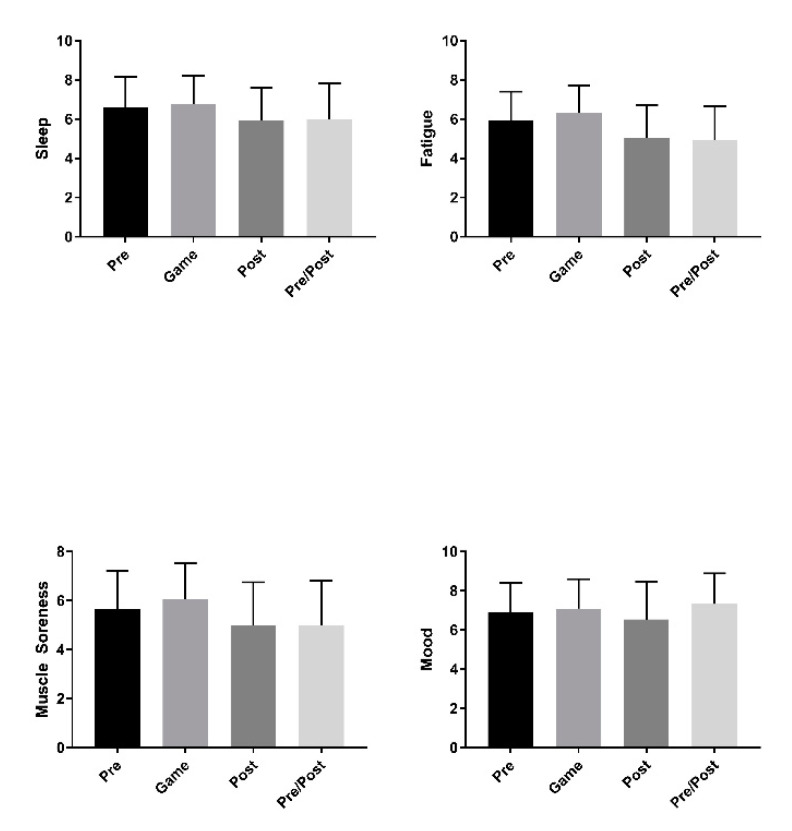
Mean (± standard deviation) athlete self-reported measures of sleep (**top left**), fatigue (**top right**), muscle soreness (**bottom left**), and mood (**bottom right**) pre-gameday, gameday, post-gameday, and pre/post gameday.

While neither HR nor HRV exhibited any significant change over the days surrounding events, sleep seemed to be negatively impacted in a small, but statistically significant way (β = −0.31 (95% CI: −0.44, −0.17), *p* < 0.001). Fatigue (β = −0.42 (95% CI: −0.54, −0.3), *p* < 0.001), muscle soreness (β = −0.33 (95% CI: −0.46, −0.2), *p* < 0.001), and mood (β = −0.14 (95% CI: −0.26, −0.01), *p* = 0.04) all showed significant negative trends following the game and/or on back-to-back game days (pre/post gameday) (Figure 3).

## 4. Discussion

The focus of the present study was to identify the potential differences in HRV across student-athletes across the varying eligibility classifications. In addition, this study aimed to highlight any possible differences in HRV between athletes of differing positions, while also documenting the HRV responses to playing at home or away and the mornings before and after soccer games. Results from this study suggest male collegiate soccer players exhibit a similar cardiac autonomic response to games throughout the collegiate season, regardless of eligibility classification or position played. Of note, despite no difference in HRV before or after competing, HRV was significantly lower when competing at home.

While HRV has been examined across a range of collegiate sports [33,34], with emphasis on collegiate soccer [35,36,37,38], very little research has focused on student-athlete eligibility classification as a factor when documenting HRV in collegiate athletes [38]. Recently, Edmonds and colleagues found that female freshman volleyball players reported lower HRV compared to all other eligibility classifications over a collegiate season, despite similar HRV responses to games and similar perceived fatigue [38]. This would suggest that female freshman athletes may carry a heavier psychological load compared to their sophomore, junior and senior student-athlete counterparts. In contrast, results from the current study found no difference in HRV across eligibility classifications in a squad of male soccer players. This lack of difference may be attributed to a different perception of stress between male and female college students. Previous research has shown that female college students experience higher levels of depression, frustration and anxiety compared to male college students [39], while also experiencing more stressors related to academic demands and relationships compared to their male counterparts [40]. As such, given the similarity in HRV across eligibility classifications in the current study, it could be argued that the different perception of stress experienced by male student-athletes may not be enough to elicit a reduction in HRV. Further, given the ASRM were also similar, it would suggest male student-athletes across all eligibility classifications perceive the various stressors of their sporting workload in a similar manner. Likewise, male student-athletes may also view the stress associated with balancing their academic and sporting workload in a similar way, without influence of their collegiate eligibility.

Somewhat surprisingly, there was no difference in HRV between pre-gameday, gameday and post-gameday measures, regardless of game location. While it has been well documented that high-intensity exercise reduces cardiac parasympathetic recovery [21,41,42], potentially up to 24–48 h after threshold intensity exercise [20], it is also well understood that individuals with enhanced cardiovascular fitness also tend to recover quicker [16,20]. With soccer considered a high-intensity intermittent sport and players reporting an average cardiovascular workload around 80–90% HRmax [25,26], it would be expected that HRV be diminished the morning after a game. However, given HRV is often quicker to recover back to within resting levels in highly trained individuals [20], it suggests the athletic cohort in this study was well trained and well prepared to physiologically handle the cardiovascular workload of games played either away or at home.

Interestingly, HRV was significantly lower during the morning of home games when compared to away games. When paired with no change in perceived stress or mood state, these results suggest the athletes displayed a greater physiological ‘readiness to compete’ for home games, compared to when competing away. Previous research has shown a link between reduced HRV and pre-competition anxiety [43,44,45,46]. However, given a similarity in self-reported stress and mood state between home and away games, it is likely that athletes in the current study were not anxious and rather were ready to compete. Indeed, lower levels of HRV prior to competition have been linked with enhanced performance and are indicative of a readiness to perform [16]. Further, early research has shown that athletes competing at home often report higher self-confidence and self-efficacy, while also reporting lower anxiety when compared to competing on the road [46,47]. These previous observations support the notion that athletes in the current study exhibited a reduced HRV at home, likely as an indication of their readiness to compete paired with an increased self-confidence.

It should be noted that, despite the potential limitations of a small sample size, the current sample (n = 17) is representative of a typical collegiate soccer squad. These findings would suggest, however, that any effect related to eligibility status is likely small and difficult to detect given the small numbers that comprise a Division I soccer team. Further, information relating to average playing time based on eligibility or position was not made available. As such, it is not known whether playing time may have contributed to the varying HRV responses across the eligibility classifications and/or positions played. Future investigation examining how playing time and starting/substitute status, when coupled with eligibility classification, impact cardiac autonomic function will shed further light on the unique nature of collegiate sport.

Given the unique physiological characteristics of soccer training and competition, future research is warranted to better apply the information within the current study to other collegiate sporting populations. Further, with student-athletes often exposed to multiple stressors associated with their academic and sporting commitments, coaches and support staff may benefit from both subjective (ASRM) and objective (HR/HRV) assessment throughout the collegiate season. Understanding how student-athletes respond to the demands imposed on them may allow coaches to better manage their athletes’ workload over the course of the season, and ultimately enhance performance. Lastly, recognizing that male student-athletes may respond differently to the stressors associated with the demands of academic and sporting workloads compared to their female counterparts may allow athletic departments to better proactively manage student-athlete wellbeing.

## Figures and Tables

**Table 1:** Participant characteristics and anthropometric measurements.

	Age (Years)	Height (cm)	Weight (kg)	Body Fat (%)
Squad (n = 17)	20.8 ± 1.0	178.4 ± 7.6	75.8 ± 3.5	6.5 ± 1.6
Freshman (n= 4)	19.8 ± 0.9	179.7 ± 9.6	75.6 ± 4.3	5.9 ± 0.6
Sophomore (n = 3)	20.0 ± 0.0	173.6 ± 7.7	74.5 ± 1.7	5.6 ± 0.6
Junior (n = 5)	20.8 ± 0.4	177.8 ± 4.8	76.5 ± 1.5	6.0 ± 0.5
Senior (n = 5)	22.0 ± 0.0	180.9 ± 8.9	76.1 ± 5.4	7.8 ± 2.5

**Table 2:** Mean (± standard deviation) heart rate and heart rate variability across eligibility classifications for all athletes across the collegiate soccer season.

Measure	Freshman (n = 4)	Sophomore (n = 3)	Junior (n = 5)	Senior (n = 5)
Heart Rate (bpm)	56.8 ± 7.4	63.7 ± 8.4	53.8 ± 8.6	63.8 ± 7.3
Heart Rate Variability	93.3 ± 9.1	84.3 ± 9.1	92.2 ± 7.9	81.6 ± 10.8

## Data Availability

The data presented in this study are available on request from the corresponding author. The data are not publicly available due to IRB-imposed restrictions.
